# In this month’s Bulletin

**DOI:** 10.2471/BLT.20.000920

**Published:** 2020-09-01

In editorials this month, Ren Minghui et al. (582) note gaps in access to medicines for noncommunicable diseases and mental health conditions. Amiya Bhatia et al. (583) call for prevention of violence against children associated with school closures and movement restrictions.

Tatum Anderson (586–587) reports on shortages of medical oxygen needed to treat people hospitalized with severe COVID-19. Melanie Saville talks to Gary Humphreys (588–589) about the challenges in developing and distributing future vaccines for SARS-CoV-2 vaccines.

## Afghanistan, Algeria, Cambodia, Democratic Republic of the Congo, Ethiopia, Gaza Strip, Iraq, Myanmar, Niger, Pakistan, Togo, Somalia, Sudan, Syrian Arab Republic, 

### Amputations, cerebral palsy, polio sequelae 

Cornelia A Barth et al. (599–614) characterize users of rehabilitation services in post-conflict settings. 

## China

### Suppressing transmission

Haijiang Lin et al. (632–637) detail COVID-19 control strategies in Taizhou city. 

## Malawi, South Africa, Zambia, Zimbabwe 

### Genital schistosomiasis, HIV and cervical cancer

Dirk Engels et al. (615–624) propose an integrated research agenda. 

## Global

### Improving the yield of mass testing for SARS-CoV-2 

Andreas Deckert et al. (590–598) compare strategies for pooled-sample analysis.

### Managing interruptions to research

Jerome Amir Singh et al. (625–631) detail effects on participants. 

### Consequences of smallpox eradication 

Rebecca Grant et al. (638–640) discuss models showing epidemic potential for monkeypox. 

### Getting the right medicine

Hannah Kettler et al. (641–643) enumerate interventions needed to improve access.

